# Enterovirus D68 Subclade B3 Circulation in Senegal, 2016: Detection from Influenza-like Illness and Acute Flaccid Paralysis Surveillance

**DOI:** 10.1038/s41598-019-50470-z

**Published:** 2019-09-25

**Authors:** Amary Fall, Ndack Ndiaye, Mamadou Malado Jallow, Mamadou Aliou Barry, Cheikh Saad Bou Touré, Ousmane Kebe, Davy Evrard Kiori, Sara Sy, Mohamed Dia, Déborah Goudiaby, Kader Ndiaye, Mbayame Ndiaye Niang, Ndongo Dia

**Affiliations:** 1 Institut Pasteur de Dakar, Département de Virologie, Dakar, Senegal; 2 Institut Pasteur de Dakar, Unité d’Epidémiologie des maladies infectieuses, Dakar, Senegal

**Keywords:** Viral epidemiology, Viral infection

## Abstract

Following the 2014 outbreak, active surveillance of the EV-D68 has been implemented in many countries worldwide. Despite subsequent EV-D68 outbreaks (2014 and 2016) reported in many areas, EV-D68 circulation remains largely unexplored in Africa except in Senegal, where low levels of EV-D68 circulation were first noted during the 2014 outbreak. Here we investigate subsequent epidemiology of EV-D68 in Senegal from June to September 2016 by screening respiratory specimens from ILI and stool from AFP surveillance. EV-D68 was detected in 7.4% (44/596) of patients; 40 with ILI and 4 with AFP. EV-D68 detection was significantly more common in children under 5 years (56.8%, p = 0.016). All EV-D68 strains detected belonged to the newly defined subclade B3. This study provides the first evidence of EV-D68 B3 subclade circulation in Africa from patients with ILI and AFP during a 2016 outbreak in Senegal. Enhanced surveillance of EV-D68 is needed to better understand the epidemiology of EV-D68 in Africa.

## Introduction

Enterovirus D68 (EV-D68) belongs to species D within *Enterovirus* genus of the *Picornaviridae* family. Following first identification in 1962, EV-D68 circulated at low levels associated with mild respiratory infections for decades, with increasing clusters of EV-D68 respiratory disease reported from 2008–2010 in Asia, Europe, and the US^1^. In 2014, the largest, most widespread outbreak of EV-D68 was reported in North America associated with considerable morbidity and mortality; including 1,153 confirmed infections and 14 deaths in the US between August and December^2,3^. Importantly, EV-D68 circulation in the US in 2014 also coincided with an outbreak of acute flaccid myelitis (AFM) with 120 cases reported from 34 US states^4–6^. Although causality was not yet clearly established, the association between EV-D68 and AFM has become more clear since 2014^5–7^. A neonatal mouse model of EV-D68 myelitis^8^ adds important evidence of biologic plausibility supporting causality.

After the 2014 outbreak, active surveillance of the EV-D68 was implemented in many countries in Asia, Europe and America. This surveillance has demonstrated continued circulation of the EV-D68 and associated AFM detected outbreaks in the United States^9,10^, France^11^, Spain^12^, Netherlands^13^, Denmark^14^, Sweden^15^, Taiwan^16^ and Italy^17^. Although outbreaks of EV-D68 in 2014, 2016 have been reported in many parts of the world, EV-D68 circulation during these outbreaks remains largely unexplored in Africa except in Senegal, where low-level circulation was detected during the 2014 outbreak^18^. EV-D68 has not been previously reported in association with AFP or AFM cases in Africa, despite previous studies conducted in Gambia, Senegal^19^, Kenya^20,21^ and South Africa^22,23^.

We investigate the genetic diversity, frequency and molecular epidemiology of EV-D68 in Senegal from June-September 2016 by screening respiratory specimens from ILI surveillance and stool specimens from AFP surveillance at the Department of Virology of Institute Pasteur of Dakar

## Results

From June to September 2016, 537 nasopharyngeal swabs from patients with ILI and 59 fecal specimens from AFP patients were screened for EV-D68 **(**Table 1**)**. The age of patients ranged from one month to 69.4 years (mean 10.9 years, median 3.6 years). More than half of patients tested (55.4%; 330/596) were less than 5 years old and 15.6% (95/596) were over 50 years old. The male/female ratio of tested patients was 1.07.

**Table 1:** Demographic, clinical characteristics and detection of 14 patients infected with enterovirus D68 from June to September 2016.

	Sample tested N (%)	EVd68 positive N (%)	p-Value
N (%)	596 (100)	44 (7,4)	
ILI	537 (90,1)	40 (90,9)	0,55
AFP	59 (9,9)	4 (9,1)	
**Sex**			
Female	303 (50,8)	20 (45,5)	0,28
Male	281 (47,1)	23 (52,3)	
Missing	12 (02)	01 (02,3)	
Median age (years)	3.6	3.5	0,52
**Age group (years)**			
[0–5[	331 (55,5)	25 (56,8)	**0**,**016**
[5–10[	71 (11,9)	11 (25)	
[10–15[	41 (6,9)	03 (6,8)	
[15–20[	23 (3,9)	03 (6,8)	
[20–50[	25 (4,2)	0	
[50 + [	95 (15,9)	02 (4,5)	
Missing	10 (1,7)	0	
**Clinical sign**			
Fever	557 (93,5)	40 (100)	0,16
Cough	430(80,1)	33(82,5)	0,43
Rhinitis	96(17,9)	7(17,5)	0,57
Headache	7(17,5)	60(11,2)	0,14
Pharyngitis	89(16,6)	7(17,5)	0,5
Vomiting	5(12,5)	40(7,4)	0,16
Diarrhea	3(7,5)	30(5,6)	0,39
Myalgia	02 (05)	76(14,2)	0,057

EV-D68 was detected in 7.4% (44/596) of patients: 7.5% (40/537) of ILI cases and 6.8% (4/59) of AFP cases. Among the ILI EV-D68-positive samples, 11 were in co-detection with at least one other respiratory virus. The most common co-infection was with adenovirus (8 cases), followed by influenza A (2 cases) and enterovirus (1 case). All attempts to isolate EV-D68 with standard WHO isolation procedures were negative.

Symptoms in ILI patients with EV-D68 detected included fever (100%; 40/40), cough (82.5%; 33/40), rhinitis and headache (17.5%; 7/40); with no statistically significant difference compared to EV-D68 negative ILI cases **(**Table 1**)**. All EV-D68 positive AFP patients had paralysis of one or both lower limbs, accompanied by fever at the onset of illness. Respiratory signs were not collected in the AFP surveillance form.

Overall, EV-D68 positive patients’ ages ranged from 1 month to 59.5 years (median 3.5 years). Detection of EV-D68 was significantly more common in children under 5 years compared to others age-groups (56.8%; 25/44, p = 0.016). No sex difference was seen amongst those with EV-D68 infection.

**Figure 1:**
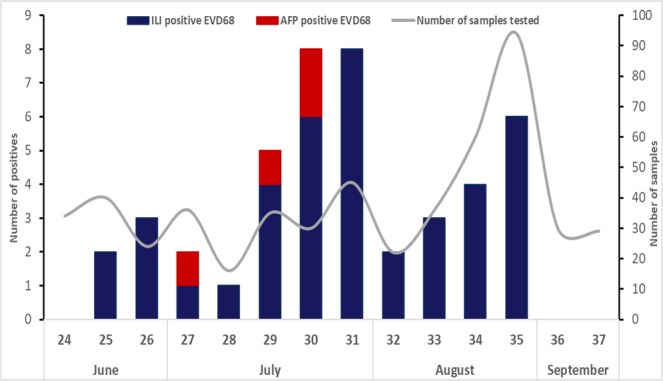
Distribution of EV-D68 in Senegal, from June to September 2016. The unbroken line represents the number of specimens collected per weeks. The shaded bars show EV-D68-positive specimens from patients with ILI (in blue) and AFP (in red).

**Figure 2:**
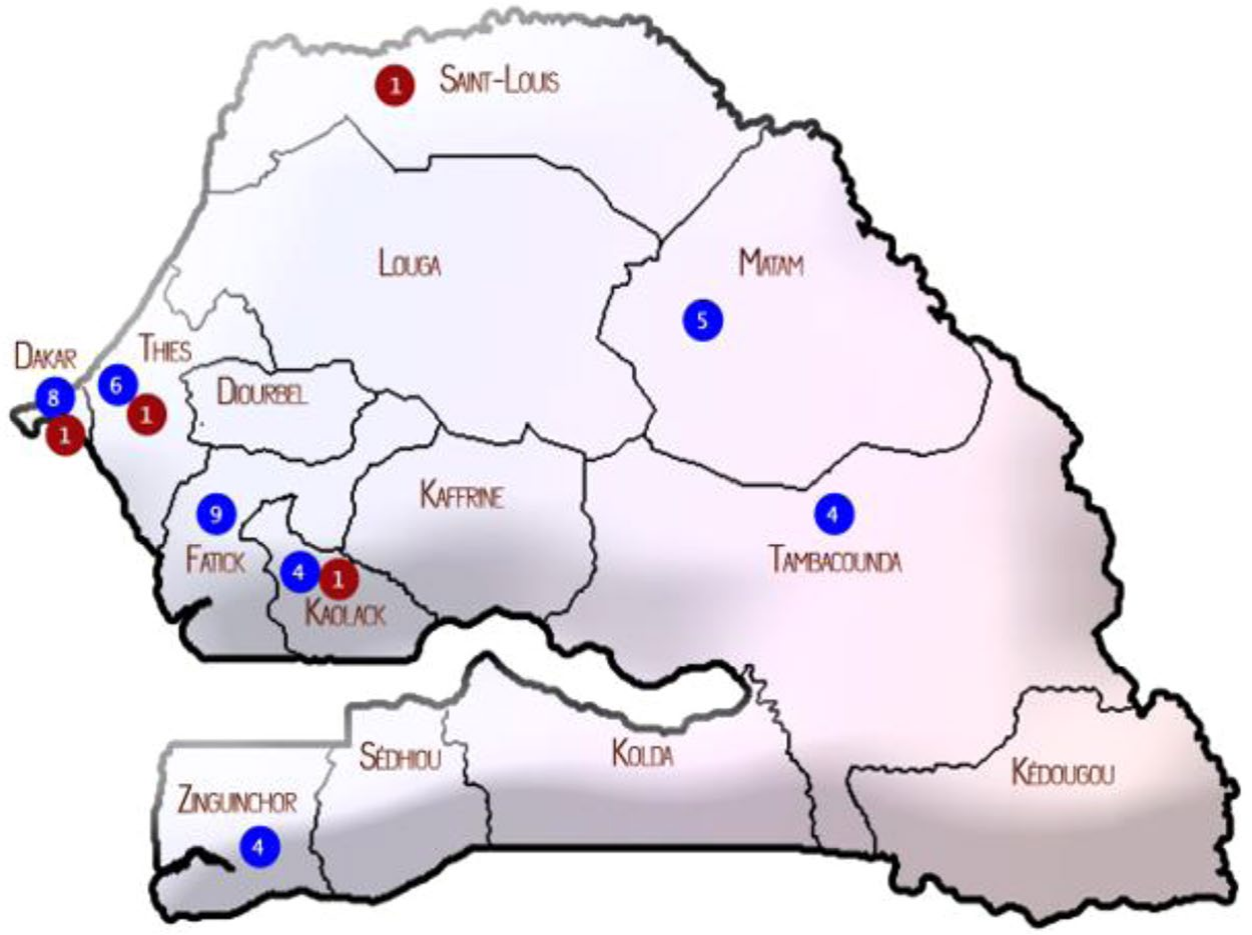
Geographical distribution of EV-D68 in Senegal with red dots representing strains from AFP patients, blues dots strains from ILI patients, the number of EV-D68 positive sample are written in dots.

EV-D68 cases were detected between week 25 and week 35, peaking during weeks 30 and 31 in July (50%; 22/44) **(**Fig. 1**)**. Patients infected with EV-D68 came from different areas in Senegal with the most cases registered in the capital city, Dakar (20.4%; 9/44), and Fatick (20.4%; 9/44) **(**Fig. 2**)**.

**Figure 3:**
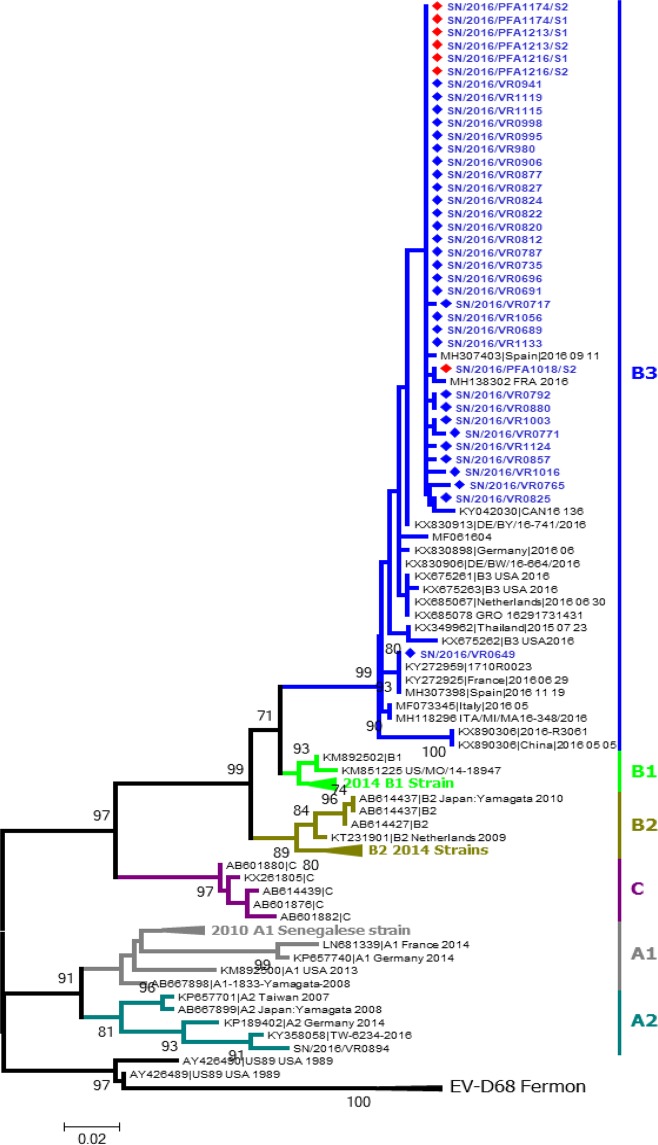
The partial sequences of VP1 region of EV-D68 were analyzed and used for a maximum likelihood phylogram. The phylogenetic tree was constructed by maximum likelihood estimation method with 1,000 bootstrap replicates using MEGA 7.0 software. The evolutionary distances were derived using the Tamura 3 parameter model. Numbers at nodes, which indicate bootstrap support values (≥70%), are given. Sequences in GenBank were also included in the analysis. Strain name, country of origin, year of detection and accession numbers are shown for each strain. Sequences of EVD68 strains from Senegal are depicted in blue with the red dots symbolizing strains from AFP patients and the blue ones strains from ILI patients.

A total of 38 sequences of the VP1 region (~800pb), including 31 from nasopharyngeal swabs and 7 from stool samples, were obtained on molecular analysis and deposited into GenBank (accession numbers MK521591-628). All strains belonged to subclade B3 and clustered with 2016 EV-D68 isolates from Spain, France, USA, Canada, Italia, Netherlands and 2015 EV-D68 isolates from Thailand and china **(**Fig. 3**)**. Within subclade B3, the Senegalese EV-D68 strains had 99.0–100% nucleotide homology with 2016 EV-D68 strains from Spain, France and Canada. There were few differences between EV-D68 sequences from ILI and AFP, with a maximum of 3 nucleotides changes. In addition, one Senegalese EV-D68 from an AFP case had 99.3% nucleotide homology with a French EV-D68 strain from an AFM case.

## Discussion

In recent years, EV-D68 circulation has been detected throughout the world, but little was previously known about EV-D68 epidemiology in Africa. We previously confirmed circulation of EV-D68 in Senegal during the large 2014 North America outbreak^18^, and this report now confirms EV-D68 circulation in Senegal during the 2016 outbreak in both AFP and ILI cases.

From June to September 2016, EV-D68 was detected in 7.4% of tested samples, including 40 ILI patients and 4 AFP patients. The period of EV-D68 circulation in Senegal correlated with EV-D68 circulation previously reported in the USA^6,9,10^, Europe^11–13,15,17,24^, Taiwan^16,25^ and in Argentina^26,27^. Regarding the timeline, as noted in other countries like USA^10^, Netherlands^13^ the first case of EV-D68 infection in Senegal was reported in June (week 25). However, earlier EV-D68 circulation has been reported in April in Italia^17^ or in March in Spain^12^.

The 7.4% EV-D68 detection rate in Senegal was lower than rates during the same time period in USA (29.1%)^10^ and Sweden (14.5%)^15^ but higher than in France (2.9%)^24^ or in Italy (2.9%)^17^.

This study provides the first report of EV-D68 detection from patients with AFP in Senegal, similar to EV-D68 detection in AFM patients in Europe^28^, Latina America^26,27,29^, Asia^16,25^ and the USA^30–33^. However, detection of EV-D68 from stool does not prove a causal relationship with paralytic disease, though all samples were negative for poliovirus.

Most of EV-D68 detections were reported predominantly in children, consistent with observations in France^24^ or in Netherlands^13^. Additionally, the median age (3.6 years) of EV-D68 infected group noted here was in line with the median age observed in Japan^34^ in 2015 and in Europe^28^ in 2016. This might be explained by small airways of young children which makes them prone to develop severe symptoms or increased susceptibility due to lack of previous exposure and protective serotype-specific immunity.

In Senegal, EV-D68 infections were detected between June and August (weeks 25–35) with more than half of cases in July (24/44) with a detection peak in weeks 30–31. This EV-D68 detection peak mapped in July in Senegal agree with findings from Netherlands^13^ while peaks were observed in August for USA^10^, June (week 26) for France^24^, September (week 35) for Sweden^15^.

This study reports the first detection of EV-D68 strains from the newly defined subclade B3^35^ in Africa. Following its discovery, EV-D68 subclade B3 had been detected in a first^36^ wave in Hong Kong^37^, Taiwan^38^, and secondary in Europe^13,15,17,24^, Latina America^26^, US^10^ and in Asia^16^ in 2016. In USA^10^, EV-D68 subclade B3 was associated with more severe respiratory illness in pediatric patients requiring intensive care unit admission compared to subclade B1. In addition, compared to the 2014 EV-D68 outbreak primarily driven by subclade B1, the 2016 subclade B3 outbreak was associated with a higher number of EV-D68-associated AFM cases in Europe with 29 cases versus 4 cases in 2014^28^. This could suggest that the circulating B3 subclade is more neuropathogenic or maybe more transmissible than the B1 clade.

This study did have some limitations inherent to retrospective screening of specimens from ILI and AFP surveillance. First, the inclusion of the fever in cases definition likely underestimates EV-D68 infections during the study period as not all EV-D68 infections lead to fever^38^. The second main weakness is the non SARI cases investigation in this study. Indeed, inclusion of all hospitalized patients in the screening would probably give a more accurate picture of EV-D68 circulation. Clinical data was collected retrospectively from ILI and AFP surveillance, therefore limited information on disease outcome was included, and atypical clinical symptoms were not reported.

This study provides the first confirmation of EV-D68 circulation in Africa in 2016 corresponding with outbreaks in the US and Europe, provides the first report of EV-D68 subclade B3 detection in Africa, and provides the first reports of EV-D68 detection from AFP cases in Africa. These findings warrant implementation of enhanced surveillance of EV-D68 respiratory infection and AFP in African countries for a better understanding of its epidemiology and burden.

## Methods

### Study population, samples and data collection

This study involved retrospective testing of respiratory and stool samples collected as part of the surveillance activities of the National Influenza Center and the WHO-accredited regional reference polio laboratory in Senegal, both hosted in the Virology Department of Institute Pasteur Dakar (IPD).

Respiratory specimens were collected from patients presenting with ILI to sentinel sites in the 4 S surveillance network^39^. A nasopharyngeal swab was collected from patients meeting the ILI case definition, placed in universal viral transport medium (Becton Dickinson and company, Italy), stored at 4–8 °C and transported to the IPD within 72 hours of collection for testing. A standardized data collection tool for ILI surveillance was used to collect demographic and clinical information.

Stool specimens were collected from patients presenting with AFP as part of routine poliomyelitis surveillance activities during the study period. Polio surveillance relies on laboratory-supported acute flaccid paralysis (AFP) case detection and confirmation, with specimens collected from AFP cases and sent to the WHO-accredited regional reference polio laboratory for processing according to the standard procedures of the WHO (Polio Laboratory Manual). Per WHO recommendations, all AFP cases under 15 years of age are reported immediately and investigated within 48 hours, with two stool specimens collected between 24–48 hours apart within one month of the onset of paralysis^40^.

### RNA extraction and real time reverse transcription-PCR (rRT-PCR)

RNA was extracted from 200 μl of nasopharyngeal swab or clarified stool suspensions (pre-treated to chloroform) using a QIAmp Viral RNA Mini Kit (QIAGEN, Hilden, Germany) according to the manufacturer’s specifications. RNAs were eluted with 60 μl nuclease-free water and stored at −80 °C until use.

All specimens from ILI and AFP patients collected from June to September 2016 were screened for EV-D68 by rRT-PCR as previously described^41^. The AgPath-ID ^TM^ one-step quantitative RT-PCR kit (Thermo Fisher Scientific, USA) was used according to the manufacturer’s instructions. Respiratory specimens were tested for respiratory viruses using the Anyplex RV16 (Seegene). Fecal specimens were inoculated onto RD cells after chloroform treatment for EV isolation according to the procedures described in the laboratory manual for the WHO Global Polio Laboratory Network^40^.

### EV-D68 Molecular characterization and phylogenetic analysis

RNA extraction and cDNA synthesis was performed as previously described^39^. The VP1 region was amplified by a Nested PCR as previously described^19^ and sent for Sanger sequencing to Genewiz (Essex, United Kingdom).

The sequences obtained in Fast format were cleaned with the GeneStudio software (GeneStudio™ Pro, version: 2.2.0.0, 8/11/2011) and Basic Local Alignment Search (BLAST) homology search program used to measure sequence matching. Sequences alignment and phylogenic analyses were performed using the MEGA 7.0 with respectively MUSCLE and ML programs. The robustness of the ML tree was assessed by bootstrap analyses of 1,000 replicates, using the 1962 Fermon strain to root the tree. The evolutionary distances were derived using the Tamura 3 parameter method. Bootstrap replicates with values ≥70 are shown on the trees.

### Statistical analysis

Statistical analyses were performed using R software (R.3.0.1 version). Continuous variables were analysed by using Mann-Whitney test, and categorical data were analysed by using the chi-square test or Fisher exact test. A P value < 0.05 was considered statistically significant.

### Ethical considerations

This study is a component of the 4S network syndromic surveillance^39^. Principles of the 4S network were approved by the Senegalese National Ethics committee hosted to the Ministry of Health in its guidelines for ILI surveillance and the Global Polio Eradication Initiative surveillance and policy. Samples collected with the objective of surveillance were approved for molecular epidemiology studies for other pathogens. For the surveillance activities, written consent was judged not necessary by the Senegalese National Ethics Committee, which has also previously approved the work of both the National Influenza Center and the National poliovirus laboratory.

## Data Availability

All data generated or analyzed during this study are included in this manuscript.
